# Effect of Gestational Diabetes Mellitus on Newborn Hearing: A Systematic Review

**DOI:** 10.1177/00034894241287014

**Published:** 2024-10-02

**Authors:** Komal Aggarwal, Rohit Ravi

**Affiliations:** 1 Department of Audiology and Speech-Language Pathology, Amity Medical School, Amity University Haryana, Gurugram, India; 2 Department of Audiology & Speech Language Pathology, Kasturba Medical College, Mangalore, Manipal Academy of Higher Education, Manipal, India

**Keywords:** newborn hearing, gestational diabetes mellitus (GDM), hearing loss

## Abstract

**Objective:**

Gestational diabetes mellitus (GDM) is associated with several adverse health conditions in newborns such as preterm birth, hyperbilirubinemia, macrosomia, respiratory distress. However, the effect of GDM on the hearing sensitivity of newborns is still unclear. The study aimed to explore the effect of GDM on newborn hearing. The study aimed to explore the effect of GDM on newborn hearing.

**Method:**

A systematic search was conducted using PubMed, Scopus, and CHINAL databases. Keywords like “gestational diabetes,” “diabetic pregnancies,” “hearing loss,” “hearing impairment,” and “hearing disorder” were used to form a search string. The Rayyan software was used for screening procedure. The full-length articles were shortlisted, extracted, and appraised.

**Results:**

The 7 articles were included in the review. Findings suggest that hearing loss is more prevalent in newborns with GDM pregnancies than in non-GDM pregnancies. In addition, OAE findings were “referred during the first hearing screening of newborns with GDM pregnancies.” The refer rate of the first bilateral hearing screening was higher for newborns with GDM pregnancies. Furthermore, children of diabetic pregnancies were found to be at risk of bilateral hearing loss, particularly sensorineural in nature.

**Conclusion:**

The present systematic review suggests an association between GDM and a higher refer rate in hearing screening. A multidisciplinary collaboration between gynecologists, pediatricians, and audiologists can smoothen the early detection of hearing loss in newborns with GDM pregnancies, leading to early intervention and better clinical outcomes to improve the quality of life of affected newborns.

## Introduction

Gestational diabetes mellitus (GDM) is defined as sugar intolerance during pregnancy and usually occurs during the 24th to 28th week of pregnancy.^1^ The GDM is characterized by severe insulin resistance and placental hormonal release. GDM can be categorized as A1GDM and A2GDM The A1GDM type is responsive to nutritional therapy and managed without medication. On the other hand, A2GDM is treated with medication to establish appropriate glycemic control.^2^ The global prevalence of GDM is 14.7% based on the International Association of Diabetes and Pregnancy Study Groups (IADPSG) criteria, a widely used screening procedure.^3^

The effect of GDM can increase the risk of adverse health outcomes for mothers and newborns. Mothers with GDM pregnancies are more likely to develop preeclampsia, polyhydramnios, placenta previa, gestational hypertension, and chances of preterm delivery.^4,5^ Health conditions like preterm birth, hyperbilirubinemia, macrosomia, respiratory distress, neurologic impairments, and cardiac disorders are associated with newborns of GDM pregnancies.^6^ Newborns who suffer from these health conditions are more prone to be unhealthy; thus, they require specialized tests and exclusive monitoring to determine comprehensive wellness.

Congenital hearing loss is prevalent globally and increasing gradually. In 2020, the prevalence of hearing loss was 1.8 per 1000 babies screened for hearing loss.^7^ The key infective causative agents of congenital hearing loss are TORCH (Toxoplasmosis-Others-Rubella-Cytomegalovirus-Herpes simplex), lymphocytic choriomeningitis, Zika virus.^8^ The other risk factors are neonatal intensive care of more than 5 days, exposure to ototoxic drugs, hyperbilirubinemia, low birth weight, asphyxia, hypoxia, and GDM.^9,10^ In addition, the prevalence of hearing loss in premature babies was 1.2% to 7.5% and 1.4% to 4.8% in babies weighing 750 to 1500 g.^11^ Moreover, a case report has documented a diagnosis of ipsilateral hearing loss accompanied by hypoplasia of the internal auditory canal of the ear in a newborn with maternal diabetes.^12^ One more study showed a higher failure of hearing screening in newborns with pregestational diabetes.^13^ It appears doubtful that deformity of ear and higher failure rate of hearing screening are unrelated to GDM. However, the conclusive impression of GDM on newborn hearing screening is still unknown.

These findings raise major concerns as congenital hearing loss is often associated with children’s affected quality of life, limited social and cognitive development.^14^ Importantly, the impact of untreated hearing loss affects self-esteem, less academic achievements followed by limited employment opportunities.^15^ The aim of this systematic review is to understand the effect of gestational diabetes mellitus on newborn hearing.

## Method

This systematic review was performed and reported according to the Preferred Reporting Items for Systematic Reviews and Meta-Analyses (PRISMA) statement.^16^ The protocol of this review in the International Prospective Register of Systematic Reviews (PROSPERO; registration number CRD42023465428, 10th October 2023). A systematic search was conducted using 3 electronic databases including PubMed, Scopus, and CHINAL. Keywords like “gestational diabetes,” “diabetic pregnancies,” “hearing loss,” “hearing impairment,” and “hearing disorder” with Boolean operators “AND,” and “OR” were used to formulate a search string. The Population Intervention Comparison Outcome Study Design Timeline framework was considered to select the inclusion and exclusion criteria. Where, *Population*: Women suffered from gestational diabetes, *Intervention*: Newborns with congenital hearing loss, *Comparison*: No comparison was included in this review, *Outcome*: Studies exclusively focus on the effect of GDM on the hearing of newborns and children. All study designs were selected for inclusion criteria except randomized controlled trials and case studies. The Studies published from 2000 to 2023 were considered. Articles focusing on mother’s health were excluded. Studies published in grey literature were not considered.

The Rayyan software was used to arrange the obtained studies. Initial screening procedure of obtained record was performed using the Rayyan software. Followed by duplicated removal and title screening, the remaining studies were shifted to Excel spreadsheet to check eligible studies. After abstract reading, full articles were retrieved and reviewed by both authors to make a final decision. Disagreements between both the authors were settled through discussion. The finalized studies which met the inclusion criteria proceeded for data extraction. A data extraction sheet was designed to illustrate characteristics of selected studies including publication details (eg, author, country of study), aim, study design, population details (eg., sample size, age of participants), and outcomes (key findings related to the effect of GDM on newborn hearing). Full-length selected studies were assessed for quality assurance with the help of the “Quality Assessment Tool for Observational Cohort and Cross-Sectional Studies” of the National Institute Health (NIH).^17^ To remove the chance of bias, both authors decided to review each study independently.

## Results

**Figure 1.**
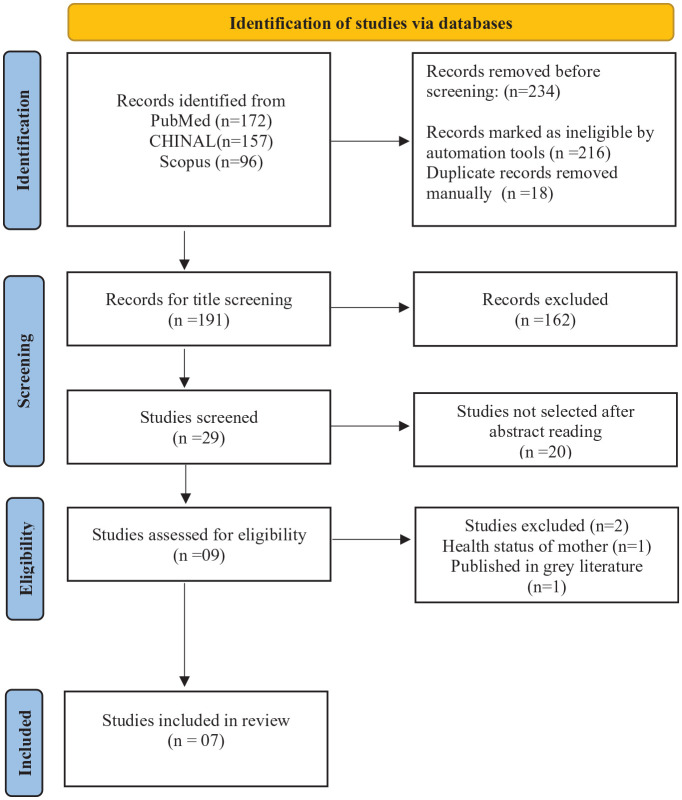
PRISMA flowchart showing the article selection process.

The search discovered 425 studies. After duplicate removal, title screening and abstract reading were performed. Nine articles were evaluated for eligibility. A total of 7 studies met the inclusion criteria published from 2020 to 2023. Figure 1, PRISMA chart indicates the process of study selection comprising abstract screening, full-text reading, and exclusion criteria.

Three studies were conducted in India, rest of the studies were carried out in the USA, China, Philippines, and Turkey. Table 1 represents publication details, aim, study design, population, and key findings of included studies.

**Table 1.** Represents Key Characteristics and Findings of Included Studies.

S. no.	Authors	Year	Country	Aim	Study type	Population	Outcomes
1.	Lee et al^18^	2020	USA	To find out characteristic of hearing loss and associated risk factors within diabetic pregnancies.	Retrospective study	311 children of diabetic pregnancies and 102 995 children of non-diabetic pregnancies.	Children with diabetic pregnancies were at more risk of bilateral hearing loss. Significantly, moderate to profound sensorineural hearing loss with high frequency was affected.
2.	Padmadasan et al^19^	2021	India	To find out prevalence of hearing loss related to diabetes.	Cross-sectional study	120 newborns. 63 boys and 57 girls.	Prevalence of hearing loss associated with diabetes was 4.16%. 10% newborns failed initial screening test, OAEs. On second stage of newborn hearing screening, 4.16% newborns failed ABR. Bilateral hearing loss reported in 3, unilateral in 2 and one had moderate sensorineural hearing loss.
3.	Zhou et al^20^	2021	China	To identify the impact of GDM on outcome of newborn hearing screening.	Retrospective study	666 women 69 women with GDM 597 without GDM	4.35% newborns of GDM group failed the hearing screening.
4.	Gulen Yıldız et al^21^	2022	Turkey	To investigate the relation between GDM and hearing loss in newborns.	Prospective study	114 newborns. 49 with GDM 65 without GDM 70 boys and 44 girls.	20 (40.8%) newborn in GDM group failed hearing screening (transient evoked otoacoustic emissions and automated auditory brainstem response). Only 5 (7.7%) newborns without GDM failed hearing screening test. Bilateral hearing screening failure was more in GDM group.
5.	Carlos-Hiceta et al^22^	2021	Philippines	To estimate the incidence rate of “refer” results of hearing screening in newborns with maternal diabetes and an association between both.	Retrospective study	150 newborns.	40% of newborns with diabetic mothers have first hearing screening (TEOAEs) referred. In contrast, only 7.9% of the newborns of non-diabetic mothers had referred results of initial hearing screening. Chances of initial hearing screening “refer” result was higher for newborns with diabetic mothers when compared to non-diabetics.
6.	Samanth et al^23^	2022	India	To identify whether preeclampsia and GDM were indicators of cochlear damage and sensorineural hearing loss in newborns.	Longitudinal Study	1068 newborns. 539 boys and 529 girls.	In GDM group, 15.8% newborns reported bilateral absent DPOAEs and 1.7% reported unilateral absent DPOAEs.
7.	Sharma et al^24^	2023	India	To search relation between GDM and congenital hearing loss and also determined prevalence of hearing loss affected by GDM.	Prospective cohort study	120 newborns 60 with GDM 60 without GDM	Newborns born with GDM showed referred OAEs findings and 13.3% failed ABR testing.

### Quality Assessment of Included Studies

The “Quality Assessment Tool for Observational Cohort and Cross-Sectional Studies” of the National Institute Health (NIH)^17^ was used to assess the quality of the included studies. Each of the studies was marked “yes,” “no,” and “not reported.” Both authors assessed each of the studies independently. If any disagreement was raised, it was resolved by discussion. Studies were assessed on the following questions. 1: Was the research question or objective in this paper clearly stated; 2: Was the study population clearly specified and defined; 3: Was the participation rate of eligible persons at least 50%; 4: Were all the subjects selected or recruited from the same or similar populations, 5: Was a sample size justification provided. The questions were responded using “yes,” “no,” and “no response.” Findings of quality assurance are depicted in Table 2.

Out of 7 studies, 6 studies^18-23^ fell under fair quality, and only 1 study^24^ fell under the good category.

**Table 2.** Quality Assessment of Included Studies.

Study ID	Q1. Research question or objective clearly stated	Q2. Study population clearly specified and defined	Q3. Participation rate of eligible persons at least 50%	Q4. Subjects selected or recruited from the same/similar populations	Q5. Sample size justified	Quality
Lee et al^18^	Y	Y	Y	Y	NR	Fair
Padmadasan et al^19^	Y	N	Y	Y	NR	Fair
Zhou et al^20^	Y	Y	Y	Y	NR	Fair
Gulen Yıldız et al^21^	Y	Y	Y	Y	NR	Fair
Carlos-Hiceta et al^22^	Y	Y	Y	Y	NR	Fair
Samanth et al^23^	Y	Y	Y	Y	NR	Fair
Sharma et al^24^	Y	Y	Y	Y	Y	Good

Abbreviations: N, no; NR, not reported; Y, yes.

## Discussion

This systematic review was conducted to comprehend the effect of GDM on the hearing acuity of newborns. A total of 7 studies met the inclusion criteria. Out of 7 studies, 3 studies represent findings from the Indian population, and rest 3 studies were performed in the USA, China, Philippines, and Turkey. The objective of this article is to bring attention to the possibility of hearing loss in newborns with GDM, encouraging clinicians to consider detailed audiological evaluations as part of the comprehensive care for these cases.

The 2 studies estimated the prevalence of hearing loss in children with GDM. Almost 71% of children with diabetic pregnancies had hearing loss, whereas only 45% of children without diabetic pregnancies exhibited poor hearing sensitivity.^18^ Another study by Padmadasan et al^19^ predicted the prevalence of hearing loss in newborns with GDM at 4.16%, which is greater than the prevalence of hearing loss in newborns without any risk factors. Studies have reported that poor gestational glycemic control can cause vascular damage in the developing inner ear.^25,26^ Furthermore, 1 more study suggested that increased insulin-like growth factor-1 can modify cochlear morphogenesis.^27^ Children with diabetic pregnancies were more likely to have high-frequency hearing loss with a severity of moderate to profound and sensorineural in nature.^18^ These conjectures indicate that children with GDM pregnancies may have impaired hearing sensitivity.

The 5 studies evaluated hearing loss in newborns by administrating otoacoustic emissions (DPOAEs and TEOAEs) and auditory brainstem response. All 5 studies found a higher failure rate of newborn hearing screening in newborns with GDM pregnancies group.^18-21,24^ Inadequate blood glucose levels during pregnancy can have harmful effects on pregnancy outcomes. A previous study examined the mechanism of oxidative stress and suggested that hyperglycemia-induced oxidative stress can interfere with proteins, fats, and deoxyribonucleic acids and cause cell damage and inaccurate organogenesis.^28^ It has been revealed that GDM may result in sensorineural hearing loss in newborns because of immunological mechanisms, microcirculation, and ischemia. The earlier studies highlighted GDM as a risk factor for poor newborn hearing screening results.^29,30^ As previously mentioned, GDM pregnancies enhance the risk of respiratory distress, hyperbilirubinemia, and metabolic disorders in newborns. Thus, it is essential to consider this alliance as an alarm to conduct hearing screening in newborns with GDM pregnancies.

## Conclusion

The results of this systematic review highlighted that GDM may affect the hearing sensitivity of newborns. The studies observed significant failure of newborn hearing screening. This is evidenced by abnormal findings of otoacoustic emissions and auditory brainstem responses that newborns with GDM pregnancies may have reduced or abnormal hearing acuity. Therefore, hearing evaluation should be mandated in newborns with GDM pregnancies to detect hearing loss at the initial stage. In addition, there is an alarming need for longitudinal studies with larger sample sizes to establish a definitive conclusion. Overall, findings from ABR and otoacoustic emissions suggest that auditory functioning may be impaired due to GDM. Additionally, healthcare professionals including gynecologists, pediatricians, and audiologists should be aware of this correlation as they can counsel the parents and family members about the importance of newborn hearing screening in GDM cases. Conclusively, it may be helpful to conduct the hearing screening in newborns who were born with GDM complications. It will accelerate the identification of hearing loss leading to a better intervention plan.
